# Green extraction optimization of triterpenoid glycoside-enriched extract from *Centella asiatica* (L.) Urban using response surface methodology (RSM)

**DOI:** 10.1038/s41598-021-01602-x

**Published:** 2021-11-11

**Authors:** Wachiraporn Thong-on, Thanika Pathomwichaiwat, Suthida Boonsith, Wanida Koo-amornpattana, Sompop Prathanturarug

**Affiliations:** 1 Department of Pharmaceutical Botany, Faculty of Pharmacy, Mahidol University, Bangkok, Thailand; 2 Department of Chemical Engineering, Faculty of Engineering, Mahidol University, Nakhon Pathom, Thailand

**Keywords:** Drug discovery, Plant sciences, Environmental sciences, Chemistry

## Abstract

*Centella asiatica* (L.) Urban extracts are widely used as food, drugs and cosmetics, and the main active compounds are glycosides (madecassoside and asiaticoside) and aglycones (madecassic acid and asiatic acid). Green extraction is an interesting concept that can produce safe and high-quality extracts that use less solvent, time and energy with the environmental friendly. This study investigated the optimum conditions for extracting a triterpenoid glycoside-enriched *C. asiatica* extract using microwave-assisted extraction (MAE) and ultrasound-assisted extraction (UAE). Central composite design and response surface methodology (RSM) were used for the experimental design and data analysis. Four-month-old *C. asiatica* tetraploid plants were selected as the elite raw material containing high amount of triterpenoid glycosides for the extraction experiments, and the triterpenoid content was determined by a validated HPLC method. The results demonstrated that the RSM models and equations were reliable and could predict the optimal conditions to enhance *C. asiatica* extract yield, glycoside and aglycone amounts. The percent of ethanol was the major factor that had a significant effect on *C. asiatica* yield and glycoside and aglycone content during MAE and UAE. The maximum triterpenoids content in extract; 7.332 ± 0.386% w/w madecassoside and 4.560 ± 0.153% w/w asiaticoside 0.357 ± 0.013% w/w madecassic acid and 0.209 ± 0.025% w/w asiatic acid were obtained by MAE with 80% ethanol at 100 watts for 7.5 min, whereas the optimal conditions for highest total triterpenoids extraction from dry plant was UAE with 80% ethanol, temperature 48 °C, 50 min enhanced 2.262 ± 0.046% w/w madecassoside, 1.325 ± 0.062% w/w asiaticoside, 0.082 ± 0.009% w/w madecassic acid and 0.052 ± 0.007% w/w asiatic acid as secondary outcome. Moreover, it was found that MAE and UAE consumed energy 59 and 54%, respectively, lower than that of the conventional method, maceration, in term of kilowatt-hour per gram of total triterpenoids. These optimized green conditions could be recommended for *C. asiatica* extraction for triterpenoid glycoside-enriched extracts production for the pharmaceutical or cosmeceutical industries and triterpenoids quantitative analysis in raw materials.

## Introduction

*Centella asiatica* (L.) Urban is a creeping perennial herb belonging to the plant family Apiaceae (Umbelliferae), also known by the common name Gotu kola or Indian pennywort^1^. This plant is widely used as food, drugs and cosmetics. *C. asiatica* has been used for a long time in traditional Asian medicine, especially in dermatological systems, such as for its wound healing abilities^2–4^, anti-inflammatory activity^5^, and treatment of venous insufficiency^6^. The main active compounds are triterpenoids due to their pharmacological activity, and they can be divided into two groups: glycosides (madecassoside and asiaticoside) and aglycones (madecassic acid and asiatic acid)^7^. The triterpenoid content varies over a wide range, i.e., the asiaticoside content varies from 0.4 to 8.0% w/w. The triterpenoid content is an indication of the raw material quality, which follows global regulations considered as quality control standards; the World Health Organization (WHO) monograph shows a madecassoside and asiaticoside content greater than 2%^8^ and The United States Pharmacopoeia (USP) shows this value for triterpene derivative content^9^. The Thai Herbal Pharmacopoeia (THP) indicates that the ethanol-soluble extract should have total triterpene content greater than or equal to 15%, and this content should be 24% for the water-soluble extract^10^. The use of *C. asiatica* extracts is increasing, and there is a high demand for it as cosmetic and pharmaceutical ingredients. One challenge is to produce enough high-quality plant materials to support the herbal industry. Our previous research found that the elite *C. asiatica* plant is a 4-month-old tetraploid line that produced higher phytomass and glycoside-rich content when used as a raw material for extraction^11,12^. Green extraction is a new concept to discover and design optimal extraction processes to reduce energy and solvent usage and use renewable natural materials that produce safe and high extract or product quality by using a proper strategy^13^. Moreover, this new technology can be transferred from laboratory experiments to the industrial scale. Method optimization plays an important role in the extraction process. Several parameters have been studied (e.g., solvent type, extraction temperature, extraction time, solvent–solute ratio, power); for example, methodologies using biodegradable and nontoxic solvents such as water and ethanol are being developed^14^. Two methods could possibly follow this concept: microwave-assisted extraction (MAE) and ultrasound-assisted extraction (UAE). Recently, scientific evidence has demonstrated that *C. asiatica* formulations rich in the glycosides; madecassoside and asiaticoside have been proved to possess pharmacological activities related to enhance collagen synthesis, wound healing, and skin protection^15–18^. Therefore, the objectives of the present study were to optimize extraction conditions to produce triterpenoid glycoside-enriched extracts for the pharmaceutical and cosmeceutical industry and to investigate the optimal conditions for maximum total triterpenoids amounts from dry plant. The effects of microwave-assisted extraction (MAE) and ultrasound-assisted extraction (UAE) techniques on the *C. asiatica* extract yield and triterpenoid content were investigated to create an RSM model for the prediction of optimal extraction conditions. In addition, time consuming and energy consumption of green extraction methods were compared with conventional method.

## Results and discussion

### Effects of the MAE and UAE extraction factors on extract yield.

The independent variables of MAE were the ethanol percentage (40–80%), microwave power (100–200 watts) and extraction time (5–10 min), and the independent variables of UAE were the ethanol percentage (40–80%), extraction temperature (40–70 °C) and time (30–90 min). Each of these variables affected six dependent parameters: the extract yield, madecassoside content, asiaticoside content, madecassic acid content, asiatic acid content and total triterpenoid content, and these results can be found as Supplementary Table S1 and Table S2 online. The maximum extract yield from UAE was slightly higher than that from MAE, at 41.80% and 38.60% w/w, respectively. The important factor influencing the extract yield from both MAE and UAE was identified by central composite design (CCD).

**Figure 1:**
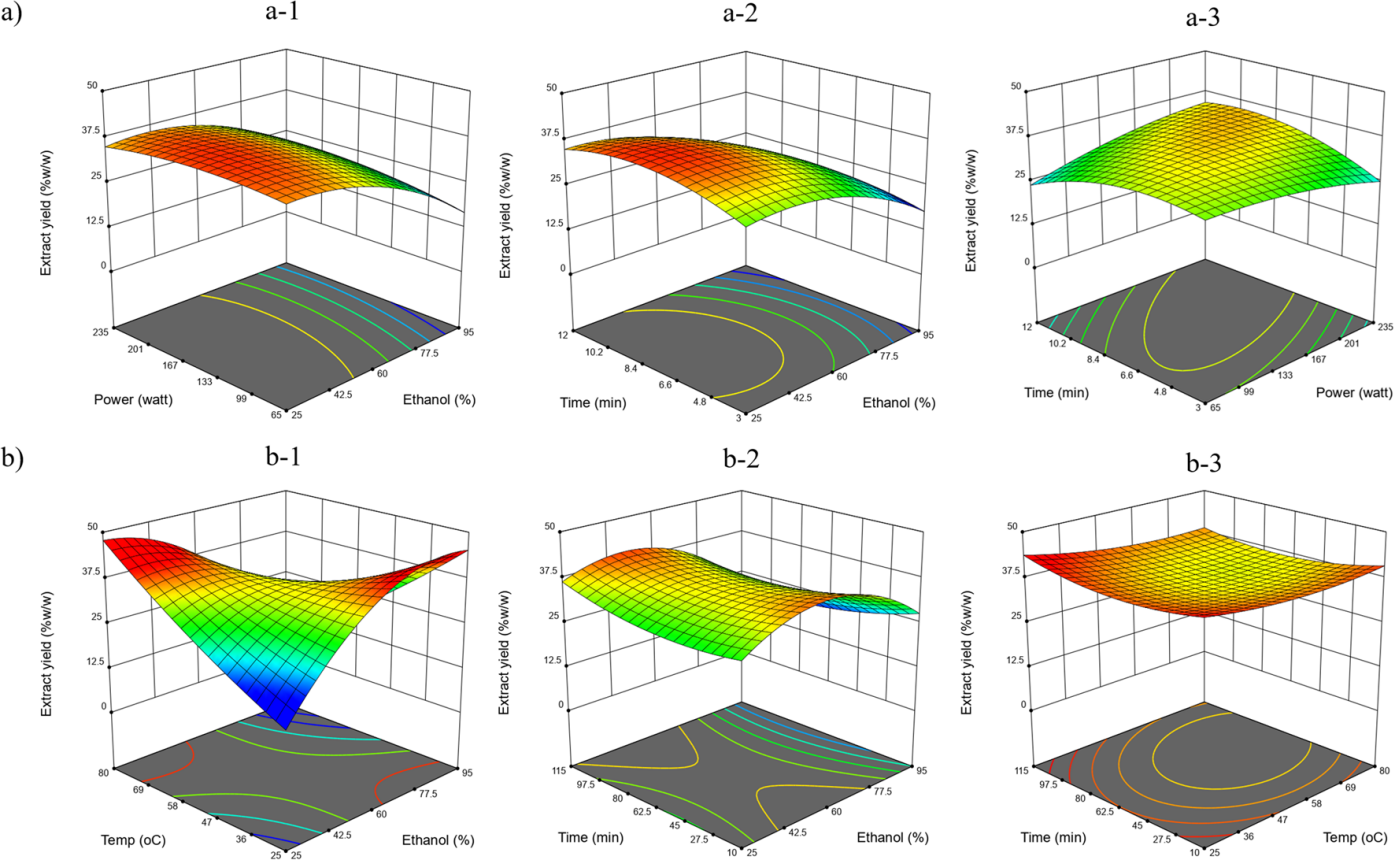
The RSM plots of green extraction conditions affect extract yield. (**a**) Microwave-assisted extraction (MAE). (**a-1**) Power and ethanol percentage at 7.5 min. (**a-2**) Time and ethanol percentage at 150 watts. (**a-3**) Time and power at ethanol concentration 60%. (**b**) Ultrasound-assisted extraction (UAE). (**b-1**) Temperature and ethanol percentage at 60 min. (**b-2**) Time and ethanol percentage at fixed 55 °C. (**b-3**) Time and temperature at ethanol concentration 60%.

Figure 1 presents the interaction effects of the independent variables on the extract yield by response surface methodology (RSM). As shown in Fig. 1a, ethanol percentage, microwave power and extraction time were observed as independent factors for MAE. The extraction yield reached its maximum at a low ethanol percentage, and the extraction time had no effect on the yield. Figure 1b presents the RSM plot of ethanol percentage, extraction temperature and time on the UAE extract yield. Similarly, to MAE, the using of a low percentage of ethanol to extract at high temperatures had a positive effect on the extract yield, and a similar result was also found at a high ethanol percentage and low temperature. Overall, the higher extract yields in both extraction methods prefers lower ethanol concentration. These results suggest that ethanol percentage and its interaction with temperature were the significant factors affecting UAE extract yield, while the main significant factors affecting MAE were ethanol percentage and the interaction of power with time (Table 1).

**Table 1:** Significant independent variables and effect on dependent parameter.

Extraction techniques	Dependent parameters	Significant independent variables^a^	Equation for coded value	R^2^
Microwave-assisted extraction (MAE)	Extract yield	%Ethanol Power*Time %Ethanol^2^ Time^2^	Extract yield = 34.89 – 4.65(%Ethanol) + 1.36(Power*Time) – 1.85(%Ethanol^2^) − 1.34 (Time^2^)	0.9688
	Glycosides			
	MS	%Ethanol Time^2^	MS = 1.90 + 0.0625(%Ethanol) – 0.0655(Time^2^)	0.6282
	AS	%Ethanol %Ethanol*Power Time^2^	AS = 1.10 + 0.0391(%Ethanol) – 0.0590(%Ethanol*Power)–0.0654(Time^2^)	0.7517
	Aglycones			
	MA	%Ethanol	MA = 0.0858 – 0.0113(%Ethanol)	0.6155
	AA	%Ethanol Time %Ethanol*Time %Ethanol^2^	AA = 0.0488 – 0.0168(%Ethanol) + 0.0040(Power) + 0.0060(Time) – 0.0028 (%Ethanol*Power) – 0.0088(%Ethanol*Time) + 0.0052(Power*Time) + 0.0154 (%Ethanol^2^) + 0.0032(Power^2^) + 0.0009(Time^2^)	0.9220
	TT	Time^2^	TT = 3.13 – 0.1277(Time^2^)	0.6766
Ultrasound-assisted extraction (UAE)	Extract yield	%Ethanol %Ethanol*Temp %Ethanol^2^	Extract yield = 36.33 – 2.58(%Ethanol) – 5.61(%Ethanol*Temp) – 3.21(%Ethanol^2^)	0.8963
	Glycosides			
	MS	%Ethanol %Ethanol*Temp	MS = 1.88 + 0.1635(%Ethanol) – 0.0876(Temp) – 0.0271(Time) – 0.3684 (%Ethanol*Temp) – 0.0651(%Ethanol*Time) + 0.0146(Temp*Time) – 0.0479 (%Ethanol^2^) + 0.0629(Temp^2^) + 0.0618(Time^2^)	0.8250
	AS	%Ethanol %Ethanol*Temp %Ethanol^2^	AS = 1.20 + 0.1076(%Ethanol) – 0.0361(Temp) – 0.0176(Time) – 0.2260 (%Ethanol*Temp) – 0.0422(%Ethanol*Time) – 0.0170(Temp*Time) – 0.1058 (%Ethanol^2^) + 0.0249(Temp^2^) + 0.0410(Time^2^)	0.9031
	Aglycones			
	MA	%Ethanol*Temp	MA = 0.0711 – 0.0194 (%Ethanol*Temp)	0.6352
	AA	%Ethanol %Ethanol*Temp %Ethanol^2^	AA = 0.0488 – 0.0220(%Ethanol) – 0.0142(%Ethanol*Temp) + 0.0132(%Ethanol^2^)	0.8292
	TT	%Ethanol*Temp	TT = 3.20 – 0.6280(%Ethanol*Temp)	0.8830

^a^Significant independent variable evaluated by ANOVA at *p* value < 0.05.

MS = Madecassoside AS = Asiaticoside MA = Madecassic acid AA = Asiatic acid TT = Total triterpenoids.

### Effects of the MAE and UAE extraction factors on triterpenoid content

**Figure 2:**
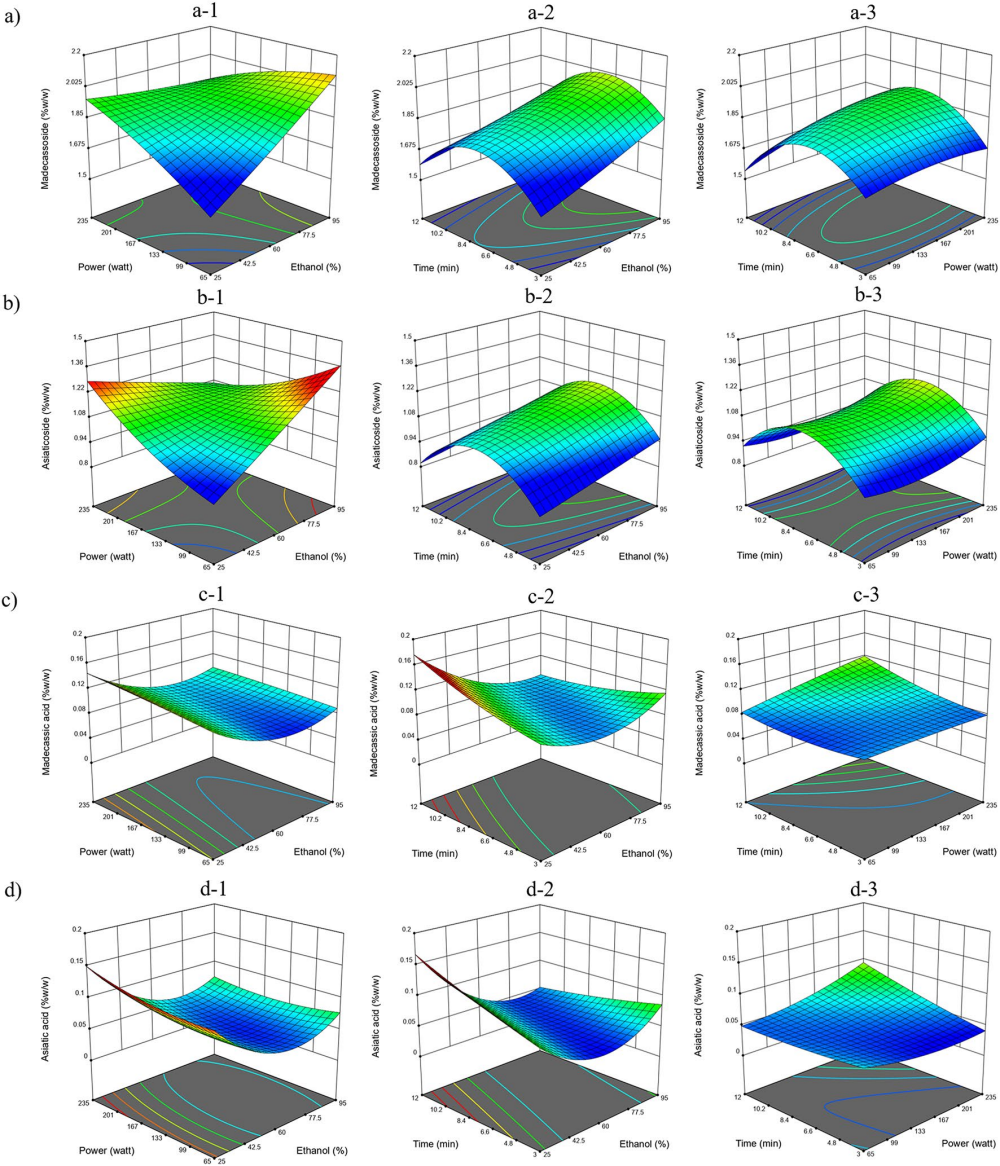
The RSM plots of MAE conditions affect triterpenoids content in dry plant. (**a**) madecassoside (**b**) asiaticoside (**c**) madecassic acid and (**d**) asiatic acid. (**a-1, b-1, c-1, d-1**) Power and ethanol percentage at 7.5 min. (**a-2, b-2, c-2, d-2**) Time and ethanol percentage at 150 watts. (**a-3, b-3, c-3, d-3**) time and power at ethanol concentration 60%.

Table 1 summarizes the significant extraction factors affecting the glycoside, aglycone and total triterpenoid contents from MAE and UAE at the 95% confidence level (*p* < 0.05). The predicted equation evaluated by statistical analysis software showed the positive or negative effects of each independent variable on the dependent parameter. For MAE, the ethanol percentage had a positive effect on the madecassoside and asiaticoside contents, while the ethanol percentage had a negative effect on the madecassic acid and asiatic acid contents (Fig. 2). The *C. asiatica* extract showed a higher glycoside content when using a higher ethanol percentage as the solvent, whereas aglycones preferred a lower ethanol percentage. The quadratic term of (ethanol percentage)^2^ displayed a positive effect on the asiatic acid content, while the time^2^ term influenced the madecassoside, asiaticoside and total triterpenoids contents with a negative effect. The interaction between the ethanol percentage and power had a negative effect on the asiaticoside content, while interaction effect on asiatic acid was between the ethanol percentage and time (Table 1). Conversely, Yingngam et al. reported that ethanol concentrations of 15 to 50% v/v enriched the extract with glycosides, whereas aglycones preferred higher ethanol concentrations (> 60% v/v)^19^. However, the other extraction parameter ranges were different: power (300–600 watts) and extraction time (0.5–5 min) could influence the solute–solvent energy conversion mechanism of the extraction process. According to the fundamentals of MAE, the heat and mass gradients flow in the same direction, heating occurs inside the solids (from the microwave-absorbed solvent) where the dissolution of the extract components takes place, and the change in the cell structure caused by the electromagnetic waves influences the extraction efficiency^14,20^. Overall, these results support that the ethanol percentage was a major factor that affected the solubility of the target compounds and that the microwave absorption ability affected the extract yield and glycoside and aglycone contents. Therefore, the optimal conditions for MAE should be a suitable point between heat and target compound transfer into the bulk solution, especially the solvent mixture conditions.

**Figure 3:**
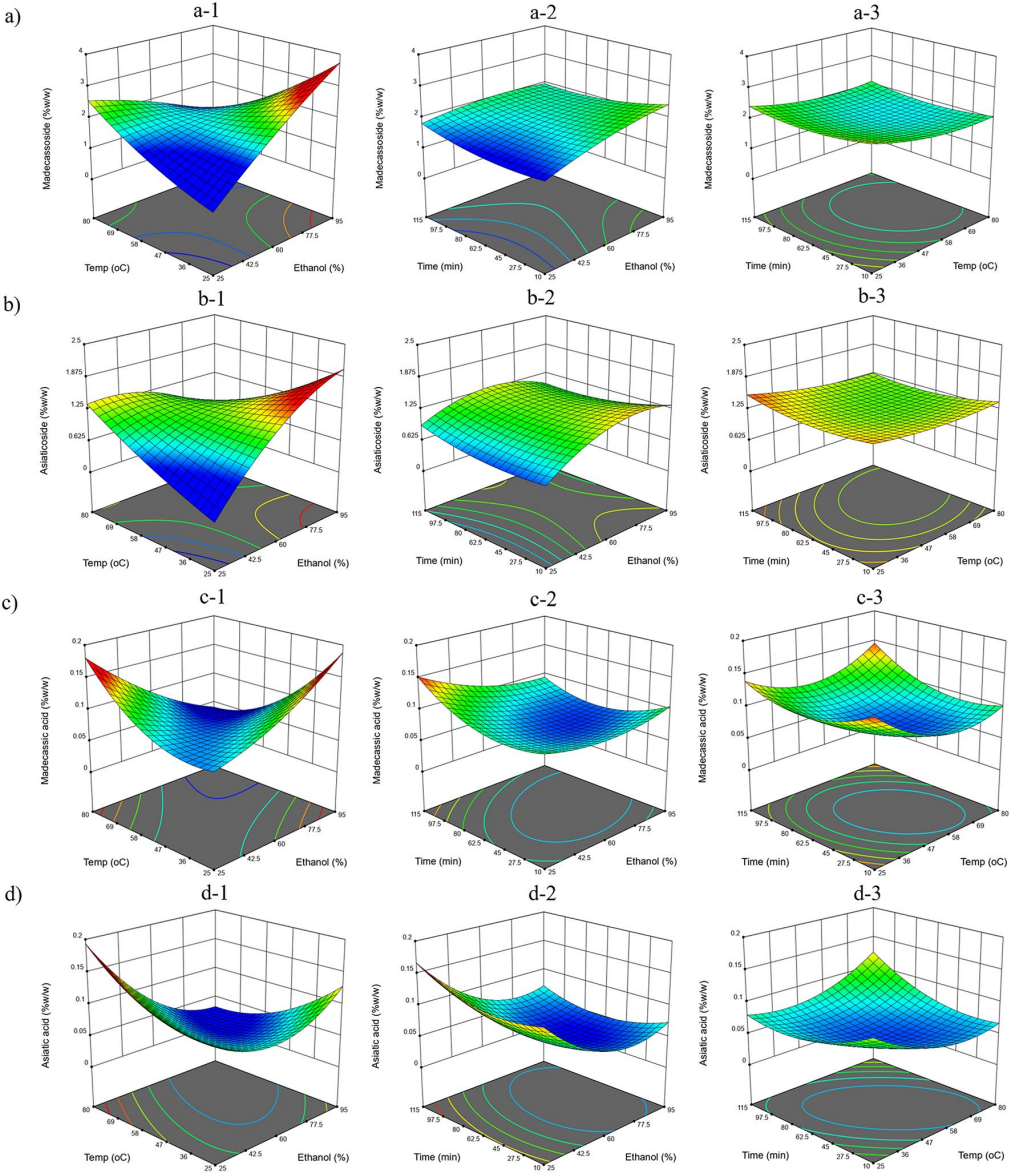
The RSM plots of UAE conditions affect triterpenoids content in dry plant. (**a**) madecassoside (**b**) asiaticoside (**c**) madecassic acid and (**d**) asiatic acid. (**a-1, b-1, c-1, d-1**) Temperature and ethanol percentage at 60 min. (**a-2, b-2, c-2, d-2**) time and ethanol percentage at 55 °C. (**a-3, b-3, c-3, d-3**) Time and temperature at ethanol concentration 60%.

For UAE, the ethanol percentage had a negative effect on the contents of asiaticoside and asiatic acid, while this term showed a positive effect on madecassoside content and had no effect on the madecassic acid content (Fig. 3). The *C. asiatica* extract showed a higher accumulation of asiaticoside and asiatic acid when using a lower ethanol percentage as the solvent, whereas madecassoside preferred a higher ethanol percentage. The term of (ethanol percentage)^2^ influenced asiaticoside and asiatic acid with different effects, while the interaction between the ethanol percentage and temperature had a negative effect on all responses (Table 1). Similarly, Seong et al. reported that when extraction temperature was fixed at 50 °C, the significant factors affected glycosides content were methanol percentage and ultrasonic power while time had no effect^21^. Energy absorption and system temperature affect the kinetic properties and dielectric constant (polarity) of the solvent especially the solvent mixed with water (e.g.water-alcohol mixture). Increasing the extraction temperature of the water results in a lower dielectric constant and less polarity. In each of the extraction conditions of the UAE and MAE the changing of energy and temperature in solvent system that may change in the solvent polarity to enhance the extraction capabilities of target compounds^22^.

### The optimal extraction conditions comparison with conventional methods and verification

As shown in Table 2, the optimal UAE parameters for *C. asiatica* glycoside extraction from dry plant determined by RSM were 80% ethanol as the solvent at a temperature of 48 °C for 50 min. The predicted values of madecassoside and asiaticoside contents were 2.280 and 1.361% w/w, respectively. For MAE, these predicted values were lower than those for UAE, 1.973% w/w for madecassoside and 1.198% w/w for asiaticoside at the optimal extraction parameters of 80% ethanol at 100 watts for 7.5 min.

**Table 2:** Predicted and experimental values of responses at optimal green extraction conditions compare with conventional method.

Methods	Constraints	Optimal extraction conditions	Values	Extract yield (% w/w)	Responses (% w/w)									
					Glycosides (GL)				Aglycones (AG)				Total triterpenoids (TT)	
					Madecassoside		Asiaticoside		Madecassic acid		Asiatic acid			
					% in dry plant	% in extract	% in dry plant	% in extract	% in dry plant	% in extract	% in dry plant	% in extract	% in dry plant	% in extract
Green methods	Maximum glycosides	*MAE* Ethanol 80% Power 100 w Time 7.5 min	Predicted	26.56	1.973	–	1.198	–	0.080	–	0.049	–	3.300	–
			Experimental	25.37 ± 1.01^c^	1.858 ± 0.065^b^	7.332 ± 0.386^a^	1.156 ± 0.019^a,b^	4.560 ± 0.153^a,b^	0.091 ± 0.003^b^	0.357 ± 0.013^a^	0.053 ± 0.005^c^	0.209 ± 0.025^b^	3.157 ± 0.044^b^	12.458 ± 0.485^a^
			Error (%)	4.48	5.83	–	3.51	–	13.75	–	8.16	–	4.33	–
			95% CI	25.05–28.07	1.847–2.100	–	1.113–1.284	–	0.057–0.102	–	0.037–0.060	–	3.096–3.504	–
		*UAE* Ethanol 80% Temp 48 °C Time 50 min	Predicted	34.26	2.280	–	1.361	–	0.084	–	0.050	–	3.775	–
			Experimental	33.97 ± 1.01^b^	2.262 ± 0.046^a^	6.662 ± 0.178^a^	1.325 ± 0.062^a^	3.905 ± 0.259^b^	0.082 ± 0.009 ^b,c^	0.240 ± 0.020^c^	0.052 ± 0.007^c^	0.152 ± 0.017^c^	3.721 ± 0.092^a^	10.960 ± 0.403^b^
			Error (%)	0.85	0.79	–	2.65	–	2.38	–	4.00	–	1.43	–
			95% CI	31.51–37.00	2.068–2.492	–	1.258–1.464		0.062–0.106	–	0.033–0.068	–	3.493–4.058	–
	Maximum aglycones	*MAE* Ethanol 40% Power 153 w Time 10 min	Predicted	37.17	1.775	–	1.000	–	0.102	–	0.097	–	2.974	–
			Experimental	35.81 ± 1.53^a,b^	1.652 ± 0.095^c^	4.623 ± 0.374^b^	1.031 ± 0.155^b^	2.888 ± 0.484^c^	0.114 ± 0.012^a^	0.318 ± 0.043^a,b^	0.096 ± 0.006^b^	0.268 ± 0.009^a^	2.893 ± 0.244^b^	8.096 ± 0.856^c^
			Error (%)	3.66	6.93	–	3.10	–	11.76	–	1.03	–	2.72	–
			95% CI	35.66–38.68	1.648–1.901	–	0.913–1.084	–	0.075–0.129	–	0.086–0.109	–	2.789–3.198	–
		*UAE* Ethanol 40% Temp 55 °C Time 90 min	Predicted	37.42	1.783	–	1.060	–	0.123	–	0.098	–	3.064	–
			Experimental	38.73 ± 1.30^a^	1.790 ± 0.081^b,c^	4.622 ± 0.190^b^	1.155 ± 0.035^a,b^	2.984 ± 0.085^c^	0.127 ± 0.008^a^	0.327 ± 0.025^a^	0.116 ± 0.008^a^	0.302 ± 0.019^a^	3.188 ± 0.118^b^	8.235 ± 0.281^c^
			Error (%)	3.50	0.39	–	8.96	–	3.25	–	18.37	–	4.05	–
			95% CI	34.06–40.77	1.523–2.042	–	0.934–1.186	–	0.100–0.145	–	0.077–0.120	–	2.697–3.389	–
Conventional method		*Maceration* Ethanol 80%, shaking for 6 h and set aside for 18 h at ambient condition	Experimental	25.25 ± 0.04^c^	1.839 ± 0.016^b^	7.284 ± 0.076^a^	1.204 ± 0.045^a,b^	4.769 ± 0.187^a^	0.066 ± 0.001^c^	0.262 ± 0.003^b,c^	0.044 ± 0.001^c^	0.175 ± 0.004^b,c^	3.153 ± 0.058^b^	12.490 ± 0.250^a^

Experimental values are presented as the mean ± standard deviation (SD) (n = 3). Different letters within the same column indicate significant different samples (DMRT, ANOVA *p* < 0.05). 95% CI = 95% confidence interval at lower and upper limit.

Compared to aglycone extraction from dry plant, the optimal UAE conditions to extract the maximum aglycones were 40% ethanol at a temperature of 55 °C for 90 min, and the predicted values were 0.123% w/w madecassic acid and 0.098% w/w asiatic acid. Similar extraction condition patterns (a low ethanol percentage and a longer extraction time) were found for MAE, with 40% ethanol at 153 watts for 10 min as the suitable conditions to enhance the predicted maximum madecassic acid and asiatic acid contents at 0.102 and 0.097% w/w, respectively. Moreover, these conditions produced higher extract yields in UAE and MAE at 37.42 and 37.17% w/w, respectively.

Several MAE studies have reported that the microwave power and time for *C. asiatica* extraction varied across the wide ranges of 180–650 watts and 0.5–30 minutes^19,23–27^. Moreover, solvent mixtures of alcohol and water at temperatures between 60 and 90 °C were most frequently applied for plant extraction^28–30^. There have been report of natural deep eutectic solvent (NADESs), modified solvent consists of acetylcholine chloride: malic acid: water (1:2:2): water (40:60) was used as optimal solvent for *C. asiatica* extract^26^. Since each report used raw materials grown under different conditions (e.g. accession, cultivation site, harvesting age), it was not possible to completely compare target compounds yields in the extracts that using same technique at different operational conditions. One of the key considerations to achieve optimal conditions is the interaction of microwave power and extraction time. The ideal condition was the factor setting for extract as much as possible target compounds without causing degradation and no solvent evaporation occurs during the extraction process. From our preliminary study, the use of a high ethanol percentage with a high power (for MAE) or high temperature (for UAE) led to solvent evaporation before the extraction processes were completed and resulted in lower extraction yields. This finding agrees with a previous study; a temperature higher than the solvent boiling point affected to the degradation of the active compounds^25^, and the longer extraction time caused a gradual decreasing in the content of heat-labile active compounds^19^.

Verification of the optimal conditions was confirmed by the experiments performed. As shown in Table 2, the predicted values of all responses in dry plant were close to the experimental values. The errors between these values for MAE and UAE were in the ranges of 1.03–13.75% and 0.39–18.37%, respectively, which were within the acceptable range of the 95% confidence interval. These results demonstrated that the RSM model and equations were reliable and could predict the optimal conditions to enhance *C. asiatica* extract yield and glycosides and aglycones contents in dry plant. Moreover, all extract yields met the requirements of the THP, which states that the ethanol-soluble extract should have greater than or equal to 15% w/w triterpenoid content. The total triterpenoid content was also within the criteria of the WHO monograph, which requires that the chemical assay of *C. asiatica* should contain more than 2% of asiaticoside and madecassoside, and the USP-required total triterpenoids should be greater than 2%.

The proportion of glycosides and aglycones from MAE and UAE after setting the constraints and running the experiments confirmed a similar pattern. For the glycosides-dominant constraints of both MAE and UAE, the ratios of glycosides to aglycones were 21:1 and 27:1, respectively. The aglycones-dominant constraints showed ratios of were 13:1 and 12:1, respectively, which resulted in higher amounts of madecassic acid and asiatic acid.

Raw material quality is the main factor that needed to be considered at the initial step before the efficiency extraction method is applied. The difference in the initial proportion of active compounds in the raw material depends on plant genetics, agricultural conditions, and harvesting and post-harvesting processes and directly affects the pre-extraction active components. In this study, tetraploid *C. asiatica* with a dominant content of glycosides was used as the starting material; therefore, all conditions showed mainly glycoside extracts with different proportions of aglycones. However, this finding demonstrated that the RSM model and prediction equations were reliable and could be applied to predict the optimal conditions to enhance *C. asiatica* extract yields and active compound contents. Conventional extraction method in this study was standard maceration conditions chosen from Thai Herbal Pharmacopoeia (THP) by using 80% ethanol as solvent at room temperature. Monton et al. reported that at high extraction temperature and time performed highest madecassoside and asiaticoside contents. Twice extraction with 95% ethanol at 60 °C for 120 min performed maximal yields of glycosides; madecassoside and asiaticoside at 0.855 and 0.174% w/w and aglycones; madecassic acid and asiatic acid 0.053 and 0.174% w/w^29^. However, triterpenoids yields were still lower than THP criteria. This may be due to the quality of *C. asiatica* starting raw materials.

Comparing the green extraction with the conventional methods, MAE and UAE showed significant different and higher extraction efficiency in terms of the yield and target compound amounts and are effectively time consuming than maceration and could be applied in different purposes. Among the green methods, results from Table 2 indicated the MAE optimal extraction conditions for product extraction with highest total triterpenoids (glycosides-enriched extract), which were 80% ethanol, 100 watts and 7.5 min, presented the total triterpenoids amounts at 12.458 ± 0.485% w/w. The crude extract contained madecassoside content (7.332 ± 0.386% w/w), asiaticoside content (4.560 ± 0.153% w/w), madecassic acid content (0.357 ± 0.013% w/w) and asiatic acid content (0.209 ± 0.025% w/w), which were within the acceptable THP criteria, as the amounts of madecassoside and asiaticoside in the extract should be more than 4% and 3% w/w, respectively. These extraction conditions could be recommended for glycoside-enriched extract production for skin benefits or anti-aging purposes in the pharmaceutical and cosmeceutical industries. *C. asiatica* extracts with a specific proportion of active compounds could guarantee the quality of the extract and products. MAE closed-vessel system reported by Shen et al., extraction with 90% methanol at 70 °C for 20 min produced a higher *C. asiatica* triterpenoid yield in a shorter extraction time than sonication and conventional methods^23^. Similarly, Puttarak et al. found that MAE enhanced triterpenoid yields more than the heat reflux method by using absolute ethanol as the solvent at high microwave power (600 watts) for four irradiation cycles (one cycle: 15 s power on and 30 s power off)^31^. Yingngam et al. reported optimal MAE conditions at lower ethanol percentage (58%), at 300 w for 3.4 min produced highest triterpenoids however the energy consumption by using high microwave power for an extraction cycle consume more energy 23% higher than our results^19^. Conversely, the optimal MAE conditions for all target compounds in our study were observed in the low-power range of 100 to 200 watts with longer extraction times of 5 to 10 min. Under these conditions, the temperature was stable and lower than the ethanol boiling point, and the extraction process ran continuously in one cycle; moreover, more triterpenoids accumulated than in UAE and the conventional method. Due to solvent in MAE vessel are heated faster and more uniformity than indirect external heating of UAE, while heating occurred inside plant cells are direct effected target compounds releasing easily in short time^14,32,33^. Overall, these results indicate that MAE demonstrated maximum triterpenoid glycosides content in extract while required lower energy consumption and time compared to UAE and conventional methods (e.g. heat reflux method and maceration).

For extraction efficiency from dry plant, Table 2 showed that the highest total triterpenoids obtained by UAE method with 80% ethanol, temperature 48 °C, 50 min, that demonstrated the extract yield (33.97 ± 1.01% w/w), madecassoside content (2.262 ± 0.046% w/w), asiaticoside content (1.325 ± 0.062% w/w), madecassic acid content (0.082 ± 0.009% w/w), asiatic acid content (0.052 ± 0.007% w/w) and total triterpenoids (3.721 ± 0.092% w/w). The extraction mechanism of UAE is different from MAE due to heat transferred from solvent (outer) to plant cell (inner), while cell wall be destroyed by sonication thus the compounds were released into solvent in different direction^32,33^. The ability of the cavitation and penetration of the solvent into the cell depends mainly on solvent physical properties (suitable for the target substances). The interaction of solvent type and temperature had directly affected the extraction efficiency. This method could be utilized in extracting triterpenoids content from *C. asiatica* raw materials for quantitative analysis as close as possible to the amount contained in raw materials and obtaining a higher value or not a statistically significant difference in comparison with conventional methods.

The comparison of time consuming and energy consumption of MAE, UAE and maceration at the same evaporating process showed in Table 3. MAE consumed extraction time less than maceration 22 times while produced total triterpenoids at 1.90 and 1.13 g, respectively. UAE extraction time was 13.67 times less than maceration while total triterpenoids were 2.23 g. Moreover, it was found that MAE and UAE at optimal conditions consumed lower energy (kWh) 59 and 54%, respectively, than that of the conventional method per gram of total triterpenoids. These results suggest that a green extraction method, MAE or UAE, could be applied for industrial scale extraction of *C. asiatica* and target compounds analysis for elite *C. asiatica* material screening.

**Table 3:** Time consuming and energy consumption of MAE and UAE compared with maceration.

Evaluation parameters	Extraction method		
	Maceration	MAE	UAE
Extraction and evaporation process of optimal conditions	Shaking (6 h) and set aside (18 h) and evaporation (1 h)	Microwave extraction (100 w for 7.5 min) and evaporation (1 h)	Ultrasonic extraction (48 °C for 50 min) and evaporation (1 h)
Equipment power (w) in extraction process	352	100	1,800
Extracted yield (g)*	15.15	15.22	20.38
Total triterpenoids (g)* in extract	1.13	1.90	2.23
Time consuming (hr)	25.00	1.13	1.83
Energy consumption (kWh)	6.81	4.71	6.20
Energy consumption(kWh) /Extracted yield (g)	0.45	0.31	0.30
Energy consumption(kWh) /Total triterpenoids (g)	6.03	2.48	2.78

*From initial dry plant material at 60 g in an experiment cycle.

## Materials and methods

### Plant material preparation

Triterpenoid glycoside-rich tetraploid *C. asiatica* were induced by our research group^11,12^, were used as the starting plant materials. Tetraploid plantlets were propagated and cultivated following previous protocols^34^. The aerial parts of four-month-old plants were harvested, washed and dried in a hot air oven at 50 °C for 24 h. The dried plants were homogenized into a powder, passed through a No. 20 sieves and kept in the dark. Plant manipulations complied with institutional, national, and international guidelines and legislation.

### Plant sample extraction

From preliminary extraction experiment, solid–liquid ratio of 1:5, 1:10 and 1:20 were studied and the results showed that ratio of 1:10 and 1:20 have no difference in yields while 1:5 performed lower yields, therefore 1:10 was chosen in this study. For MAE and UAE, ten grams of dried *C. asiatica* powder was soaked in 100 ml of solvent for an hour before the extraction. An Ethos-X microwave apparatus (Milestone, Italy) was used for the MAE experiments; the ethanol percentage (40–80%), microwave power (100–200 W) and extraction time (5–10 min) were investigated for the extraction. A radio frequency of 20 kHz was used for the UAE method, and the ethanol percentage (40–80%), temperature (40–70 °C) and extraction time (30–90 min) were studied. The parameter range of MAE and UAE were investigated before setting up conditions, the system temperature would not exceed solvent boiling point when instrument was in operation. The conventional extraction procedures from the Thai Herbal Pharmacopoeia (THP) were set as controls. Ten grams of powder was macerated with 100 ml of ethanol by shaking for 6 h and then set aside for 18 h under ambient conditions. Each extract was filtered through filter paper (Whatman No. 1) and evaporated to dryness, and the extract yield was recorded. All extracts were kept at 4–6 °C and protected from light before triterpenoid content determination.

### Triterpenoid content analysis

The extracts were diluted with methanol to a concentration of 5,000 µg/ml and filtered through a 0.45 µm filter for triterpenoid content analysis with an Ultimate 3000 Thermo® HPLC. The protocol of Thong-on^34^ was validated and used to determine the triterpenoid contents in the *C. asiatica* extracts. Method validation in this study followed the guidelines of the Association of Official Analytical Chemists (AOAC)^35^. A LiChroCART® column (LiChrospher® 100 RP-18, 250 mm × 4 mm I.D., particle size 5 µm), an acetonitrile gradient (solvent A) and 0.1% H_3_PO_4_ (solvent B) were used. The mobile phase system of solvent A varied as follows: 20–35% (10 min), 35–65% (15 min), 65–80% (5 min), 80–20% (5 min) and 20% (10 min). The injection volume was 20 µl, the flow rate was 1 ml/min and detection was performed at 206 nm. Four reference standards, madecassoside, asiaticoside, madecassic acid and asiatic acid, were mixed and prepared with methanol at five different concentrations: 12.5, 25.0, 50.0, 100.0 and 200.0 µg/ml. Each standard solution was injected in triplicate to construct a calibration curve, and a regression equation was used to determine the triterpenoid content in each sample. The triterpenoid contents were calculated as % w/w of the dry plant and % w/w of the extract.

### Experimental design

Central composite design (CCD) was used to investigate the effects of the independent variables on six responses from both the MAE and UAE experiments: extract yield, madecassoside content, asiaticoside content, madecassic acid content, asiatic acid content and total triterpenoid content in dry plant. The independent variables were coded at three levels (− 1, 0, + 1), and the coded and actual tested values of 20 experiments are shown in Table 4. The CCD quadratic equation was as follow Eq. (1):

1 $$ Y = \beta_{0} + \mathop \sum \limits_{i = 1 }^{3} \beta_{i} X_{i} + \mathop \sum \limits_{i = 1 }^{3} \beta_{ii} X^{2}_{i} + \mathop \sum \limits_{i = 1 }^{3} \mathop \sum \limits_{j = i + 1 }^{3} \beta_{ij} X_{i} X_{j} $$

where *Y* is the dependent parameter or response, *X*_*i*_ and *X*_*j*_ are the independent coded variables, *β*_*0*_ is the intercept, and *β*_*i*_, *β*_*ii*_ and *β*_*ij*_ are the regression, quadratic and interaction coefficients, respectively.

**Table 4:** Central composite design (CCD) for the independent variables of MAE and UAE.

Run	Independent variables; actual value (coded value)					
	Microwave-assisted extraction (MAE)			Ultrasonic-assisted extraction (UAE)		
	Ethanol (%)	Power (w)	Time (min)	Ethanol (%)	Temp (°C)	Time (min)
1	60 (0)	150 (0)	7.5 (0)	60 (0)	55 (0)	60 (0)
2	60 (0)	150 (0)	7.5 (0)	40 (− 1)	40 (− 1)	30 (− 1)
3	60 (0)	150 (0)	7.5 (0)	60 (0)	55 (0)	9.5 (− 1.682)
4	60 (0)	150 (0)	7.5 (0)	60 (0)	55 (0)	60 (0)
5	80 (+ 1)	200 (+ 1)	10 (+ 1)	60 (0)	80 (+ 1.682)	60 (0)
6	40 (− 1)	200 (+ 1)	5 (− 1)	60 (0)	55 (0)	60 (0)
7	60 (0)	150 (0)	3.3 (− 1.682)	60 (0)	55 (0)	60 (0)
8	93.63 (+ 1.682)	150 (0)	7.5 (0)	40 (− 1)	40 (− 1)	90 (+ 1)
9	60 (0)	150 (0)	11.7 (+ 1.682)	26.36 (− 1.682)	55 (0)	60 (0)
10	80 (+ 1)	200 (+ 1)	5 (− 1)	60 (0)	29 (− 1.682)	60 (0)
11	80 (+ 1)	100 (− 1)	5 (− 1)	40 (− 1)	70 (+ 1)	30 (− 1)
12	40 (− 1)	100 (− 1)	10 (+ 1)	80 (+ 1)	70 (+ 1)	30 (− 1)
13	40 (− 1)	200 (+ 1)	10 (+ 1)	40 (− 1)	70 (+ 1)	90 (+ 1)
14	80 (+ 1)	100 (− 1)	10 (+ 1)	80 (+ 1)	70 (+ 1)	90 (+ 1)
15	26.36 (− 1.682)	150 (0)	7.5 (0)	80 (+ 1)	40 (− 1)	30 (− 1)
16	60 (0)	66 (− 1.682)	7.5 (0)	60 (+ 1)	55 (0)	60 (0)
17	60 (0)	150 (0)	7.5 (0)	93.63 (+ 1.682)	55 (0)	60 (0)
18	60 (0)	234 (+ 1.682)	7.5 (0)	60 (0)	55 (0)	110.4 (+ 1.682)
19	40 (− 1)	100 (− 1)	5 (− 1)	80 (+ 1)	40 (− 1)	90 (+ 1)
20	60 ()	150 (0)	7.5 (0)	60 (0)	55 (0)	60 (0)

### Data analysis

Design-Expert® software version 12.0 and response surface methodology (RSM) were conducted to determine the optimal MAE and UAE conditions. Significant differences in each factor were separated using analysis of variance (ANOVA), and means were compared at a 95% confidence level (*p* < 0.05).

## Conclusions

Our findings demonstrated that the RSM model and prediction equations were reliable and could be applied to predict the optimal conditions to enhance *C. asiatica* extract yield and active compound contents. The MAE method demonstrated optimal conditions (80% ethanol, power 100 watts, 7.5 min) that produced the highest triterpenoids content in extract with 7.332 ± 0.386% w/w madecassoside, 4.560 ± 0.153% w/w asiaticoside, 0.357 ± 0.013% w/w madecassic acid and 0.209 ± 0.025% w/w asiatic acid. The UAE method delivered suitable conditions (80% ethanol, temperature 48 °C, 50 min) that extracted the highest total triterpenoids from dry plant with 2.262 ± 0.046% w/w madecassoside, 1.325 ± 0.062% w/w asiaticoside, 0.082 ± 0.009% w/w madecassic acid and 0.052 ± 0.007% w/w asiatic acid. These processes could therefore be recommended for glycoside-enriched *C. asiatica* extract production for pharmaceutical or cosmeceutical purposes and the highest total triterpenoids extraction for analysis.

## Supplementary Information

Supplementary Information.
