# Supplementary material for: Green extraction optimization of triterpenoid glycoside-enriched extract from Centella asiatica (L.) Urban using response surface methodology (RSM)
Source: Sci Rep. 2021 Nov 11;11:22026. doi: 10.1038/s41598-021-01602-x (PMC8586240; doi:10.1038/s41598-021-01602-x)
Supplement: Supplementary file 1 — Supplementary Information. [file 41598_2021_1602_MOESM1_ESM.pdf]

Supplementary Information

Green extraction optimization of triterpenoid glycoside-enriched extract from *Centella asiatica* (L.) Urban using response surface methodology (RSM)

Wachiraporn Thong-on[1], Thanika Pathomwichaiwat[1], Suthida Boonsith[2], Wanida Koo-amornpattana[2], Sompop Prathanturarug[1]*

[1] Department of Pharmaceutical Botany, Faculty of Pharmacy, Mahidol University, Bangkok, Thailand

[2] Department of Chemical Engineering, Faculty of Engineering, Mahidol University, Nakhon Pathom, Thailand

* Corresponding author: Sompop Prathanturarug (sompop.pra@mahidol.ac.th)

Table S1 Central composite design (CCD) of MAE variables and responses.

Run	Independent variables			Dependent parameters; %w/w					
	Ethanol (%)	Power (w)	Time (min)	Extract yield	Glycosides		Aglycones		TT
					MS	AS	MA	AA	
1	60	150	7.5	34.80	1.875	1.095	0.081	0.048	3.099
2	60	150	7.5	35.10	1.889	1.119	0.074	0.041	3.123
3	60	150	7.5	34.87	1.894	1.090	0.083	0.049	3.116
4	60	150	7.5	35.00	1.985	1.095	0.125	0.060	3.265
5	80	200	10	28.50	1.748	0.954	0.099	0.057	2.858
6	40	200	5	32.30	1.814	1.063	0.098	0.066	3.041
7	60	150	3.3	31.00	1.779	0.940	0.086	0.045	2.850
8	93.63	150	7.5	20.50	2.168	1.273	0.087	0.059	3.587
9	60	150	11.7	30.40	1.745	0.932	0.085	0.051	2.813
10	80	200	5	27.30	1.775	0.996	0.091	0.057	2.919
11	80	100	5	26.80	1.883	1.077	0.085	0.052	3.097
12	40	100	10	34.63	1.693	0.955	0.119	0.086	2.853
13	40	200	10	38.60	1.838	1.108	0.143	0.123	3.212
14	80	100	10	25.73	1.820	1.121	0.086	0.053	3.080
15	26.36	150	7.5	38.00	1.744	0.973	0.124	0.119	2.96
16	60	66	7.5	31.17	1.824	1.104	0.073	0.050	3.051
17	60	150	7.5	35.20	1.914	1.085	0.081	0.050	3.130
18	60	234	7.5	34.13	2.008	1.239	0.083	0.059	3.389
19	40	100	5	36.93	1.740	0.992	0.093	0.072	2.897
20	60	150	7.5	34.50	1.808	1.088	0.073	0.046	3.015
Min	26.36	66	3.3	20.50	1.693	0.932	0.073	0.041	2.813
Max	93.63	234	11.7	38.60	2.168	1.273	0.143	0.123	3.587

MS=Madecassoside AS=Asiaticoside MA=Madecassic acid AA=Asiatic acid TT=Total triterpenoids

Table S2 Central composite design (CCD) of UAE variables and responses.

Run	Independent variables			Dependent parameters; %w/w					
	Ethanol (%)	Temp (°C)	Time (min)	Extract yield	Glycosides		Aglycones		TT
					MS	AS	MA	AA	
1	60	55	60	34.20	1.838	1.166	0.074	0.048	3.126
2	40	40	30	31.83	1.629	0.910	0.102	0.102	2.743
3	60	55	9.5	38.75	1.984	1.294	0.078	0.055	3.411
4	60	55	60	36.90	1.872	1.166	0.074	0.049	3.161
5	60	80	60	39.70	2.119	1.283	0.085	0.059	3.546
6	60	55	60	39.60	2.067	1.308	0.068	0.053	3.496
7	60	55	60	35.50	1.670	1.222	0.064	0.048	3.004
8	40	40	90	32.73	1.616	0.995	0.112	0.094	2.817
9	26.36	55	60	34.06	1.506	0.603	0.065	0.097	2.271
10	60	29	60	37.07	1.928	1.243	0.085	0.057	3.313
11	40	70	30	40.47	1.941	1.286	0.118	0.110	3.455
12	80	70	30	27.27	1.742	1.013	0.065	0.041	2.861
13	40	70	90	39.57	1.998	1.235	0.153	0.149	3.535
14	80	70	90	23.77	1.527	0.861	0.073	0.051	2.512
15	80	40	30	41.80	2.892	1.609	0.119	0.072	4.692
16	60	55	60	36.28	1.881	1.200	0.072	0.049	3.202
17	93.63	55	60	20.98	1.878	1.171	0.071	0.049	3.169
18	60	55	110.4	40.17	2.021	1.311	0.080	0.057	3.469
19	80	40	90	38.60	2.630	1.457	0.117	0.071	4.275
20	60	55	60	35.20	1.959	1.139	0.079	0.050	3.227
Min	26.36	29	9.5	20.98	1.506	0.603	0.064	0.041	2.271
Max	93.63	80	110.4	41.80	2.892	1.609	0.153	0.149	4.692

MS=Madecassoside AS=Asiaticoside MA=Madecassic acid AA=Asiatic acid TT=Total triterpenoids
